# Questionnaires of self-perception poorly correlate with instability elicited by walking balance perturbations

**DOI:** 10.1371/journal.pone.0315368

**Published:** 2024-12-12

**Authors:** Andrew D. Shelton, Jessica L. Allen, Vicki S. Mercer, Jeremy R. Crenshaw, Jason R. Franz

**Affiliations:** 1 Joint Department of Biomedical Engineering, University of North Carlina at Chapel Hill & North Carolina State University, Chapel Hill, NC, United States of America; 2 Department of Mechanical & Aerospace Engineering, University of Florida, Gainesville, FL, United States of America; 3 Division of Physical Therapy, University of North Carolina at Chapel Hill, Chapel Hill, NC, United States of America; 4 Department of Kinesiology and Applied Physiology, University of Delaware, Newark, DE, United States of America; Kennedy Krieger Institute/Johns Hopkins University School of Medicine, UNITED STATES OF AMERICA

## Abstract

Rehabilitation to prevent falls should not only directly address intrinsic and extrinsic factors, but also the neuropsychology of falls to promote safe and independent mobility in our aging population. The purpose of this study was to determine the relation between falls self-efficacy and objective responses to a series of walking balance perturbations. 29 healthy younger adults and 28 older adults completed four experimental trials, including unperturbed walking and walking while responding to three perturbations: mediolateral optical flow, treadmill-induced slips, and lateral waist-pulls; and three self-reported questionnaires: Activity-specific Balance Confidence, Falls Efficacy Scale, and the Fear of Falling Questionnaire-Revised. We quantified stabilizing responses as a change in margin of stability from unperturbed walking. Older adults generally exhibited larger instability than younger adults in response to walking balance perturbations. Only the Fear of Falls Questionnaire-Revised showed an increase in perceived falls risk for older adults. We found no significant correlations for older adults between any balance perturbation response and questionnaires of self-perception. Given the disconnect between self-perceived falls risk and responses to walking balance perturbations, rehabilitation to prevent falls while maintaining mobility and independence will likely require personalized techniques that combine neuromuscular training with approaches for neurophysiological reeducation.

## Introduction

Falls in our rapidly aging population are a significant public health challenge. Only by understanding an individual’s risk for falls can we successfully deploy the personalized prescription of preventative measures to mitigate that risk. Factors that lead to an increased risk for falling can be multifactorial, with physiological risks involving intrinsic factors that can vary due to disease or biological aging or extrinsic factors such as environmental hazards. Because most falls occur during locomotor activities such as walking [1, 2], the cause-and-effect between physiological risk factors and falls are often measured using walking-related instability and/or responses to walking balance perturbations [3–7]. Alternatively, self-reported questionnaires examining fear of falling [8, 9], self-efficacy [10, 11], and balance confidence [12] are commonly used as proxy measures for falls risk [13] based upon self-perceptions. What began as a yes or no response to “Are you afraid of falling?” [14, 15] has evolved into more comprehensive and rigorous questionnaires of self-perception, such as the Fear of Falling Questionnaire-Revised [16], Falls Efficacy Scale [10, 11], and the Activity-specific Balance Confidence Scale [12]. While these questionnaires vary in length, topic, and scoring parameters, all are directed towards cataloging the neurophysiology of falls risk and how individuals perceive that risk.

Rehabilitation to prevent falls should not only directly address intrinsic (strength and balance) and extrinsic (environmental hazards) factors, but also the neurophysiology of falls to promote safe and independent mobility in our aging population [17]. Prescribing a treatment plan that accomplishes both translational goals requires a precision approach that meets the specific needs of each individual. As an extreme approach exclusively designed to prevent falls, one could restrict all activities likely to precipitate a fall or restrict one’s ability to complete those activities independently. However, this of course comes at a significant personal cost [18–22]. To simultaneously prevent near-term falls while promoting physical activity for long-term mobility, we must understand the relation between falls self-efficacy and the objective instability elicited by walking balance perturbations.

Unfortunately, there is a disconnect between balance and functional mobility and self-reported neurophysiological measures [8, 9, 23, 24]. For example, individuals reporting low self-efficacy but who present with high stability when faced with perturbations may unnecessarily limit their mobility or participation in daily activities. That decrease in physical activity has the potential to cascade into other detrimental changes, including accelerated declines in neural and motor integrity that can ultimately increase instability [25–27]. Conversely, individuals reporting high self-efficacy but who present with a low stability when faced with perturbations may purposefully participate in activities with a higher risk for falls and injury. Past research has assessed the relation between self-perception and performance of standing balance integrity and found evidence of a fundamental disconnect [28]. Moreover, Delbaere et al. (2010) showed disparities between physiological and perceived falls-risk through the use of the Falls Efficacy Scale International and a series of functional physiological tests including vision, proprioception, isometric strength, reaction time, and postural sway [24]. These foundational studies have demonstrated the disconnect between psychological and physiological balance outcomes. However, the use of walking balance perturbations are widespread in walking balance research and this disconnect between objective vulnerability and self-reported neurophysiological measures has yet to be explored in the context of walking-related instability.

Therefore, the purpose of this exploratory study was to analyze the relation between falls self-efficacy (i.e., fear, self-efficacy, confidence) and objective stability responses to a series of walking balance perturbations. Older adults are commonly presumed to have higher walking-related instability and an increased risk of falls compared to younger adults. Therefore, we first included a confirmatory hypotheses to benchmark the demographics of our study cohort. Specifically, we hypothesized that, compared to younger adults, older adults would have a lower self-efficacy. We then hypothesized that questionnaires of falls self-efficacy would be poor correlates of the effect of balance perturbations on stability, evidenced by no correlation between self-perception and stability responses.

## Methods

### Participants

Twenty-nine healthy younger adults (YA) (14 female, age(mean ± standard deviation): 22.4 ± 3.0 yrs, height: 1.73±0.08 m, mass: 67.2±8.6 kg) and twenty-eight older adults (OA) (15 female, age: 73.0±5.94 yrs, height: 1.70±0.08 m, mass: 71.6±19.6 kg) participated in this study. We included participants 18–35 years (younger adults) and over 65 years (older adults) with no leg injuries or prostheses, without neurological, musculoskeletal, or cardiopulmonary disease, and who could walk without the use of an assistive device. Five older adult participants self-reported that they had experienced at least one fall in the past year. Methods and recruitment procedures for this study were approved by the University Institutional Review Board (20–0555). Participants provided written informed consent prior to participating. Recruitment for this study began on April 15^th^, 2020 and recruitment for this research study ended on December 31^st^, 2022. This research study was completed with the same subject cohort and experimental walking trial data as a previously published study [29].

### Equipment

Retroreflective markers placed on the anterior and posterior iliac spines, sacrum, lateral femoral condyles, lateral malleoli, posterior calcanei, first and fifth metatarsal heads, acromia, 7^th^ cervical spine, 10^th^ thoracic spine, sternum, and the sternal notch plus an additional 14 tracking markers placed in clusters on the lateral thighs and shanks were recorded with a 10-camera motion capture system (Motion Analysis Corporation, Santa Rosa, California, USA) at 100 Hz. Participants completed all data collection trials walking on a dual-belt, instrumented treadmill (Bertec, Columbus, Ohio, USA).

### Self-reported questionnaires

At the start of the study session, participants completed three self-reported questionnaires including the Activity-specific Balance Confidence (ABC), Falls Efficacy Scale (FES), and the Fear of Falling Questionnaire-Revised (FFQ-R). Each questionnaire catalogs salient features of an individual’s perception relating to safely and confidently participating in daily lives. The ABC [12] is a 16-item questionnaire that asks participants to rate their balance confidence from 0 (no confidence) to 100 (absolute confidence) across a series of common activities performed during daily living. A higher average score would indicate higher confidence in their ability to complete tasks in their day-to-day life and a lower risk for falls. The FES [10, 11] is a 10-item questionnaire that asks participants to score their confidence in completing activities of daily living without falling from 1 (very confident) to 10 (not confident at all). While the FES and ABC are quite similar, they both target distinct domains of daily living. For example, the FES specifically asks about confidence with regards to preventing a fall while the ABC asks about the maintenance of balance, not necessarily a disruption of balance severe enough to precipitate a fall. Values for each item are then totaled where lower scores indicate higher confidence and a lower risk for falls. The FFQ-R [16] includes a combination of 15 statements that assess self-efficacy as well as fear of falling. The participant gives each statement a score of 1 (strongly disagree) to 4 (strongly agree). A higher total score indicates higher fear of falling and a higher perceived risk for falls.

### Protocol

The preferred walking speed for each participant (YA: 1.34±0.12 m/s, OA: 1.19±0.19 m/s) was obtained from the average of four 30-m overground walking trials timed using photocells (Bower Timing Systems, Draper, Utah, USA). Participants completed a three-minute warm-up walk before data collection to acclimate to treadmill walking but received no practice or training for any perturbation paradigm prior to the data collection. For data collection, participants completed four walking trials including one unperturbed and three perturbation trials at their preferred walking speed in a randomized order while wearing an overhead harness. In the unperturbed trial, participants walked for two minutes without exposure to balance perturbations at their preferred walking speed. The three perturbation paradigms have been previously described in depth in our previously published work on this same cohort and perturbation protocol [29]. Briefly, these perturbations included the following, all completed at participants’ preferred walking speed. Mediolateral optical flow perturbations were applied via a projection-based environment displaying a virtual hallway superimposed with pseudorandom oscillations shown previously to introduce walking instability[30–32]. Treadmill-induced slip perturbations involved exposure to 6 m/s^2^ treadmill belt decelerations lasting 200 ms applied at random heel strike events [5, 33, 34]. Lastly, lateral waist-pull perturbations involved exposure to a 5% body weight, 100ms pulling force directed towards the swing leg applied at random toe-off events [35].

### Measurements & analysis

The primary outcome measure in this study for walking stability and perturbation response was margin of stability (MoS). Further detail on the calculaton of MoS can be found in the previous work where the values were calculated [29]. MoS was extracted at heel strike of the left and right leg. Mediolateral (MoS_ML_) and anteroposterior (MoS_AP_) MoS were averaged across all strides for the optical flow perturbation trial and the unperturbed walking trials. For discrete perturbations, MoS outcomes were calculated at the instant of heel strike directly following perturbation onset (i.e., the recovery step) and then averaged across all perturbation occurrences within the trial [35, 36]. The ipsilateral limb beginning its stance phase at this instant of heel strike was used to define the base of support boundary. Averaged strides were used instead of the first occurrence for the discrete perturbations to more accurately replicate the nature of the requiste analysis for the continuous optical flow perturbations where adaptation and training may have occured. Stability responses to perturbations were quantified using the actual change in MoS (ΔMoS) from the unperturbed experimental walking trial to response to perturbations using Eq 1.


$$\Delta MoS={MoS}_{Perturbed}-{MoS}_{Unperturbed}$$ Eq 1

A positive ΔMoS is indicitative of an increase in stability, while a negative ΔMoS is indicative of a decrease in stability in response to perturbations. As the recovery step in the forward direction was used in the calculation of margin of stability, the same vector direction of stability was used for each perturbation to maintain consistency between paradigms.

### Statistical analysis

For the first hypothese, two-way t-tests tested for significant effects of age on and questionnaire scores with an alpha level of 0.05. To test our third hypothesis, we first calculated bivariate Pearson correlations between ΔMoS of each perturbation (treadmill slip, waist pull, and optical flow) and each questionnaire score (ABC, FES, and FFQ-R). This was performed for both directions (ML and AP) and for the total cohort, younger adults only, and older adults only, for 54 total correlations. As this was an exploratory analysis, corrections for multiple comparisons of correlations were not performed. For further interpretation into the disconnect between questionnaire score and instability from walking balance perturbations, 95% confidence interval ellipses were plotted.

## Results

### Scores for questionnaires of falls self-efficacy

**Fig 1:**
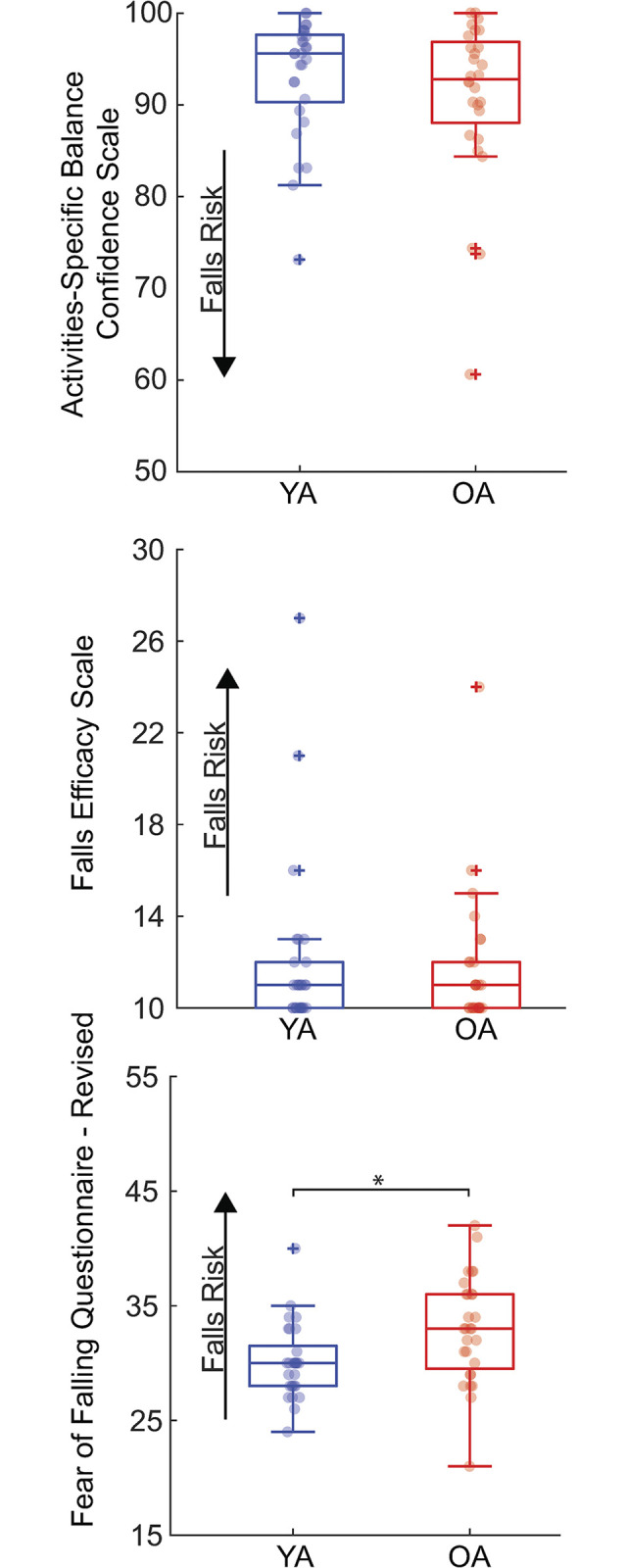
Boxplots for falls self-efficacy based on the scores for the three questionnaires: Activities-specific Balance Confidence Scale, Falls Efficacy Scale, and the Fear of Falling Questionnaire-Revised. Older adults had a significantly lower falls self-efficacy based on the results of the Fear of Falling Questionnaire-Revised compared to younger adults. Asterisks (*) indicate significant pairwise differences (p<0.05) between cohorts.

**Fig 2:**
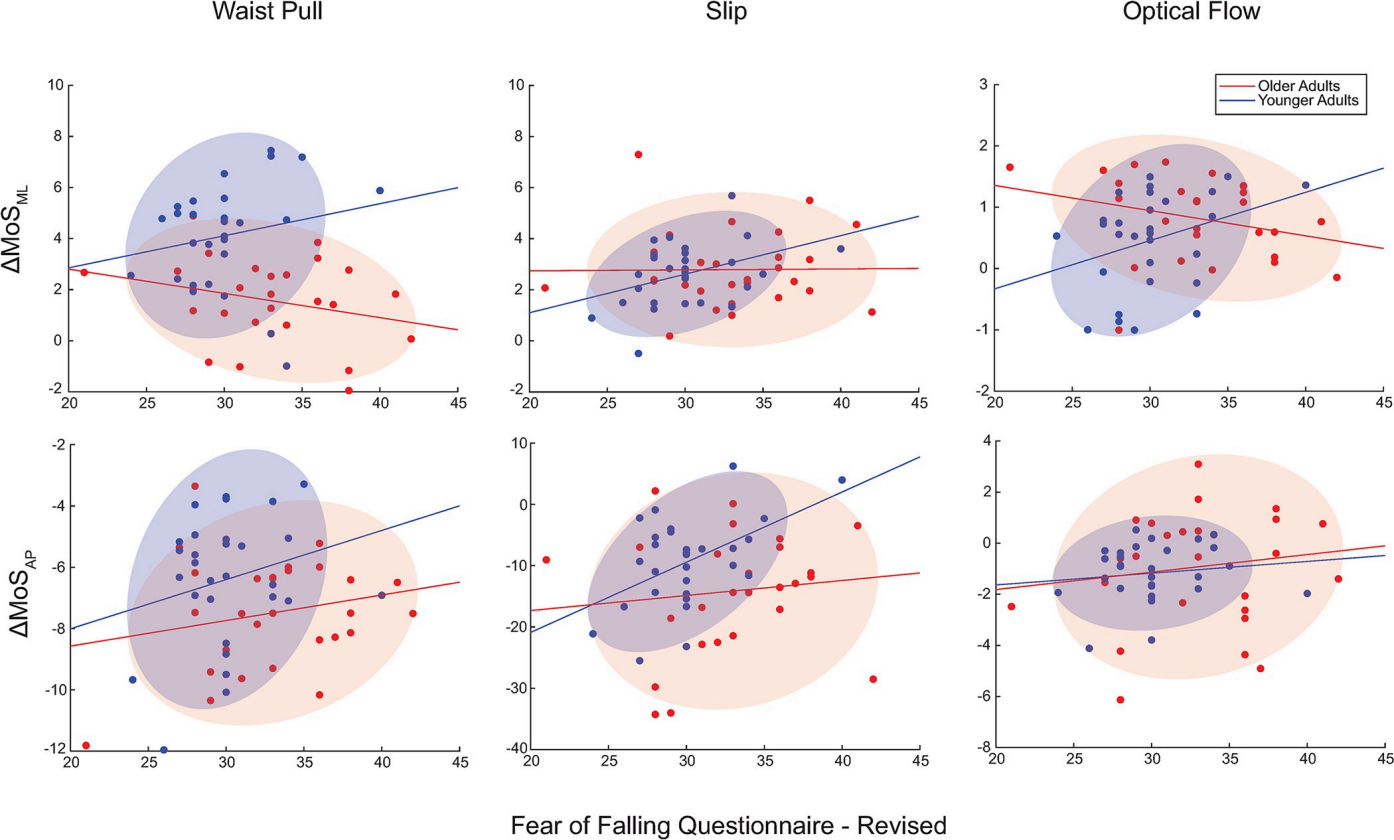
95% Confidence interval ellipses for younger and older adults for each of the perturbation responses versus the score of the Fear of Falling Questionnaire–Revised.

Only the FFQ-R showed lower falls self-efficacy for older compared to younger adults (p = 0.007, d = 3.97) (Fig 1). There were no significant differences between groups for the ABC or FES (p≥0.25) (Fig 2).

### Relationship between perceived falls risk and objective vulnerability

Out of 54 total correlations, only 5 perturbation responses correlated with measures of falls self-efficacy. These were only found in younger adults, where ML and AP responses to treadmill-induced slips correlated with FFQ-R score, ML responses to treadmill-induced slips and AP responses to optical flow perturbations correlated with the FES score, and ML responses to treadmill-induced slips correlated with the ABC score (r≥0.394, p≤0.0339) (Table 1). We found no significant correlations for older adults between any balance perturbation response and questionnaires of self-perception (r≤0.285, p≥0.145) (Table 1). We also found no significant correlations between any balance perturbation response and questionnaire of self-perception in the combined cohort of younger and older adults (r≤0.241, p≥0.070) (Table 1).

**Table 1:** Correlations between questionnaire scores and balance stability.

Activities-specific Balance Confidence					Falls Efficacy Scale				FFQ-R			
	ML		AP		ML		AP		ML		ML	
**Waist-Pull**	**r**	***p***	**r**	***p***	**r**	***p***	**r**	***p***	**r**	***p***	**r**	***p***
YA	-0.069	*0*.*724*	-0.184	*0*.*338*	0.200	*0*.*298*	0.243	*0*.*204*	0.218	*0*.*257*	0.205	*0*.*287*
OA	0.179	*0*.*363*	0.216	*0*.*270*	-0.276	*0*.*156*	0.212	*0*.*280*	-0.213	*0*.*277*	-0.145	*0*.*460*
ALL	0.136	*0*.*313*	0.070	*0*.*603*	-0.241	*0*.*070*	0.101	*0*.*454*	0.064	*0*.*635*	0.072	*0*.*593*
**Slip**												
YA	-0.428*	*0*.0210*	-0.270	*0*.*154*	0.394*	*0*.034*	0.499*	*0*.006*	0.504*	*0*.005*	0.140	*0*.*468*
OA	0.085	*0*.*667*	0.142	*0*.*473*	0.012	*0*.*954*	0.118	*0*.*549*	-0.199	*0*.*311*	-0.211	*0*.*281*
ALL	-0.116	*0*.*389*	0.035	*0*.*798*	0.164	*0*.*224*	0.131	*0*.*333*	0.158	*0*.*240*	-0.024	*0*.*861*
**Optical Flow**												
YA	-0.202	*0*.*294*	-0.257	*0*.*178*	0.323	*0*.*088*	0.132	*0*.*495*	0.055	*0*.*777*	0.395*	*0*.034*
OA	0.239	*0*.*220*	0.213	*0*.*277*	-0.285	*0*.*145*	0.145	*0*.*462*	-0.243	*0*.*213*	-0.170	*0*.*387*
ALL	0.035	*0*.*798*	0.068	*0*.*606*	0.078	*0*.*564*	0.156	*0*.*245*	-0.069	*0*.*680*	0.049	*0*.*717*

The following abbreviations are used in Table 1: FFQ-R–Fear of Falling Questionnaire-Revised, ML–mediolateral, AP–anteroposterior, YA–younger adults, OA–older adults, ALL–combined younger and older cohort, Waist-Pull–lateral waist-pull perturbations, Slip–treadmill-induced slip perturbations, and Optical Flow–mediolateral optical flow perturbations. Significant correlations are indicated through an *.

From the 95% confidence interval ellipses created from the perturbation responses (ΔMoS_ML_ and ΔMoS_AP_) and questionnaire score for both the younger and older adult cohorts, younger adults have a more condensed ellipses with a more positive sloping major axis than compared to the older adults.

## Discussion

Understanding an individual’s risk for falls through the interaction and potential disconnect between their self-perceptions and physiological risk may be important in the design of preventative measures to mitigate risk. Self-reported outcomes have been commonly used as proxy measures for falls risk while perturbation paradigms are more exclusive to research laboratories for the assessment of walking-related instability. Yet, our cumulative results show a disconnect between these neurophysiological and physiological measures of falls-risk. In partial support of our first hypothesis, the FFQ-R revealed an age-related increase in self-perceived falls risk. Finally, in support of our second and most innovative hypothesis, we found no correlations between self-perception and objective walking-related instability in our older adult cohort.

Older adults had worse stability responses than younger adults to most balance perturbation paradigms. Treadmill induced slip-perturbations revealed more negative AP responses (i.e., indicative of instability) in older adults versus younger adults, an outcome consistent the perturbation’s direction of action. Lateral waist-pulls elicited age-related differences for both the ML and AP directions. Older adults exhibited larger negative AP responses and smaller positive ML responses than younger adults to lateral waist-pulls. Both of these changes allude to smaller margins of stability in older responding to this perturbation and thus lesser stability than in younger adults. One alernative explanation for smaller ML responses in older adults could be a disproportionate use of generalized neuromuscular control in anticipation of perturbations. We have shown this phenomenon for older adults in prior research studies [31]. Although applied at random heel-strikes, the threat of a lateral waist-pull was not only ever present during that trial, but because of our experimental setup was also applied to a known direction (left or right). Older adults may view an imminent perturbation as a greater risk to their balance than younger adults and may adopt more “rigid” biomechanics and control. Williams et al. noted that older adults with greater fear of falling will stiffen their bodies when perturbed [37]. This stiffening precaution may decrease center of mass displacement and ML response for our older adult cohort that had an increased fear of falling per the FFQ-R compared to younger adults.

Unlike the more pervasive age-related differences in objective perturbation responses, only one age-related difference was found for self-perceived falls risk. Specifically, older adults in this study perceived themselves at a lower self-efficacy than younger adults only through their responses to the FFQ-R. The FFQ-R asks participants to choose a response from strongly agree to strongly disagree for a series of statements that also included activities beyond those of daily living. Conversely, the ABC and FES questionnaires ask participants to score themselves on either balance confidence or how likely they believe they would be to fall during a variety of activities; we noted saturation in both towards the lowest end of falls risk. We do not intend to call into question the veracity of the ABC and FES, particularly given the relatively healthy nature of our older adult cohort. Nevertheless, the lack of an age-related difference in these questionnaires despite objective response to walking balance perturbations does at least allude to the possibility for a disconnect between psychological measures and walking instability.

Out of 54 total possible correlations between objective response to perturbations and self-perception, we found that only 5 reached statistical significance–all in our younger adult cohort. For older adults and our combined cohort, we found no correlations between perturbation responses and any questionnaire. One possible explanation is the lack of questions across the three questionnaires that specifically target perception of falls risk with respect to reactive responses to environmental perturbations. Only the ABC includes related questions with the two activities of being bumped into by people while walking and walking outside on an icy sidewalk. Past research studies have been mixed on the ability to relate questionnaire questionnaires with balance tasks to determine falls risk. Allison et al. (2013) found that participation restriction and fear of falling (using the Survey of Activities and Fear of Falling in the Elderly, respectively) both correlated with Timed Up & Go and Berg Balance Score, but only participation restriction was able to accurately predict Timed Up & Go and Berg Balance Score ability[23]. Conversely, the results of Schinkel-Ivy et al. (2016) showed no difference between those with and without a fear of falling (via a single “yes” or “no” question) and quiet standing center of pressure or measures during unperturbed walking (speed, double support time, step length or width variability) [38]. Yet, when correlated with ABC scores, those authors identified significant positive correlations with preferred speed and double support time. Ultimately, an overview of the available literature is quite clear; correlations between self-perception and either functional correlates or standing balance seem to depend on the decision to include specific outcomes. We contend for this reason that it is critical to better understand the association between self-perception and objective walking-related instability.

To gain further insight into the disconnect between psychological measures and walking instability, we looked at 95% confidence interval ellipses of the data set including questionnaire responses and stability responses. Across the different perturbation paradigms and directionality of stability responses, older adults are seen to have a greater dispersion in their association between psychological measures and walking instability. This points to the need for personalized therapies and treating them as individuals when developing rehabilitative programming. Individuals with good stability responses and high falls self-efficacy likely have the capabilities to accommodate intrinsic and extrinsic risks while also having the requisite self-perceived ability to maintain high levels of physical activity. How we shift all individuals to this level of performance and self-perception may depend on their personal relationship between self perception and stability response. Those individuals on the other end of the spectrum, with poor stability responses and low falls self-efficacy, would likely benefit most from both balance and strength training as well as educational interventions to reduce perceived fear of falling [9]. Time and cost constraints can act as limiting factors on rehabilitative care received, so it is important to prioritize the needs of the person. Those who perceive themselves with lower falls self-efficacy, but who have smaller pertruabtion responses would likely benefit most from strategies designed to improve their independence and ability to live mobile and active lifestyles. Fear of falling and self-imposed physical activity restrictions can cause a pathophysiological cascade leading to declines in physical fitness and muscular capabilities, which can have future impacts on falls risk [39–41]. A fear of falling is prevalent in 50–70% of individuals who have experienced a fall in the past year, but 20–50% of older adults still develop this fear in the absence of a recent fall [42–45]. Those who perceive themselves with high falls self-efficacy but who have higher perturbation responses would likely benefit more from strengthening and balance training than improving self-perception and independence. We believe that this phenotype might be at the most significant risk for future falls because they may partake in activities that have a higher risk for falls. It is essential to work with the person to view this as harm reduction instead of a form of activity restriction to prevent the negative impacts that activity restriction causes [46].

There are several possible limitations that are relevant to our findings. First, the perturbation paradigms applied in this study do not currently have response magnitudes that can be directly associated with a high or low risk for falls. A key future area of research should be defining perturbation magnitudes and accompanying cutoff responses tailored to walking instability in response to balance perurbations. This work should be revisted with those defined cutoffs in mind. While certain falls-risk classification systems appear to lack efficacy in their relation to walking balance integrity, their accessibility outside of the lab is a strength. Indeed, our perturbations are also currently restrictive in access due to the space and equipment requirements. Progress to develop similar and more clinically feasible paradigms outside of the research lab environment will improve accessibility and impact. As noted previously, the lateral waist-pull perturbations in this study were applied one side at a time (left or right). This could have allowed participants to take a more cautious and planned approach to perturbations than they anticipated. As motion capture markers were not placed on the arms of our participants, a reduced set of markers of the pelvis were used to approximate center of mass. We also note that margin of stability, itself but a instantaneous biomechanical outcome related to instability, may not accurately capture true physiological falls risk, per se. In addition, perturbation magnitudes were submaximal and thus none of our participants experienced any actual falls during those trials. Despite the methodological challenges, we certainly appreciate the need for additional research designed to determine the extent to which submaximal perturbation magnitudes and the neuromuscular corrections they elicit are related to those that may precipitate actual fall events. Finally, the older adult cohort recruited for this study could be considered healthier and more mobile than the average older adult. For example, the average Dynamic Gait Index of our older adult cohort was 22.5, with scores lower than 19 points associated with impairment of gait and increased falls risk (in-depth subject demographics shown in supplementary) [47]. A more representative participant pool of all individuals could have provided greater variance in our results.

### Conclusions

We found that older adults exhibited less stable responses than younger adults to two of our three perturbation paradigms. However, only one questionnaire detected an age-related decrease in falls self-efficacy. The disconnect was also highly prevalent in a lack of significant correlations and qualitative review of our four quadrant analyses. Given the apparent disconnect between falls self-efficacy and objective responses to walking balance perturbations, rehabilitation techniques to prevent falls while maintaining mobility and independence likely requires personalized techniques to include combining balance and strengthening exercises with approaches for neurophysiological reeducation.

## Supporting information

S1 Table Subject demographic information and comparisons between the younger and older adult cohorts. (DOCX)
