# Supplementary material for: Questionnaires of self-perception poorly correlate with instability elicited by walking balance perturbations
Source: PLoS One. 2024 Dec 12;19(12):e0315368. doi: 10.1371/journal.pone.0315368 (PMC11637265; doi:10.1371/journal.pone.0315368)
Supplement: S1 Table — (DOCX) [file pone.0315368.s001.docx]

Questionnaires of self-perception poorly correlate with instability elicited by walking balance perturbations

Andrew D. Shelton^a^, Jessica L. Allen^b^, Vicki S. Mercer^c^,

Jeremy R. Crenshaw^d^, and Jason R. Franz^a^

^a^ Joint Department of Biomedical Engineering, University of North Carlina at Chapel Hill & North Carolina State University, Chapel Hill, NC, USA

^b^Department of Mechanical & Aerospace Engineering, University of Florida, Gainesville, FL, USA ^c^Division of Physical Therapy, University of North Carolina at Chapel Hill, Chapel Hill, NC, USA ^d^Department of Kinesiology and Applied Physiology, University of Delaware, Newark, DE, USA

**Supplemental Document:** This document contains extra data elements which provide a more extensive breakdown of the subject demographics.

**Supplemental Table S1** below accompanies the note in the limitation paragraph of the manuscript that the older adult population of this study could be considered healthier than average. It contains subject demographic information and two-way T-test comparisons between the younger and older adult cohorts.

**Supplemental Table 1:** Subject Demographics

|  | Younger Adults | Older Adults | P-Value | Cohen’s D |
| --- | --- | --- | --- | --- |
| Age (yrs) | 22.4 ± 3.1 | 73.0 ± 5.9 | - | - |
| Height (m) | 1.73 ± 0.08 | 1.70 ± 0.11 | 0.334 | 0.26 |
| Mass (kg) | 67.2 ± 19.6 | 71.6 ± 19.6 | 0.284 | 0.29 |
| Walking Speed (m/s) | 1.34 ± 0.12 | 1.19 ± 0.19 | <0.001 | 0.95 |
| 5 Repetition  Sit-to-Stand (s) | 14.4 ± 2.5 | 15.5 ± 4.2 | 0.121 | 0.42 |
| Dynamic Gait Index (score) | 24.0 ± 0 | 22.5 ± 2.37 | 0.001 | 0.89 |
| Strenuous Exercise (# of times per week) | 3.4 ± 2.0 | 1.3 ± 1.5 | 0.007 | 1.17 |
| Moderate Exercise (# of times per week) | 5.1 ± 2.7 | 3.2 ± 2.5 | 0.007 | 0.75 |
| Easy Exercise (# of times per week) | 5.2 ± 3.0 | 2.7 ± 2.2 | <0.001 | 0.94 |
